# Complexities of post-transcriptional regulation and the modeling of ceRNA crosstalk

**DOI:** 10.1080/10409238.2018.1447542

**Published:** 2018-03-23

**Authors:** Claire L. Smillie, Tamara Sirey, Chris P. Ponting

**Affiliations:** MRC Human Genetics Unit within the Institute of Genetics and Molecular Medicine, University of Edinburgh, Edinburgh, UK

**Keywords:** microRNA, competitive endogenous RNA, post-transcriptional regulation, RNA-induced silencing complex, cooperativity, subcellular localization

## Abstract

Control of gene and protein expression is required for cellular homeostasis and is disrupted in disease. Following transcription, mRNA turnover and translation is modulated, most notably by microRNAs (miRNAs). This modulation is controlled by transcriptional and post-transcriptional events that alter the availability of miRNAs for target binding. Recent studies have proposed that some transcripts – termed competitive endogenous RNAs (ceRNAs) – sequester a miRNA and diminish its repressive effects on other transcripts. Such ceRNAs thus mutually alter each other’s abundance by competing for binding to a common set of miRNAs. Some question the relevance of ceRNA crosstalk, arguing that an individual transcript, when its abundance lies within a physiological range of gene expression, will fail to compete for miRNA binding due to the high abundance of other miRNA binding sites across the transcriptome. Despite this, some experimental evidence is consistent with the ceRNA hypothesis. In this review, we draw upon existing data to highlight mechanistic and theoretical aspects of ceRNA crosstalk. Our intent is to propose how understanding of ceRNA crosstalk mechanisms can be improved and what evidence is required to demonstrate a ceRNA mechanism. A greater understanding of factors affecting ceRNA crosstalk should shed light on its relevance in physiological states.

## Graphical abstract

**Figure:**
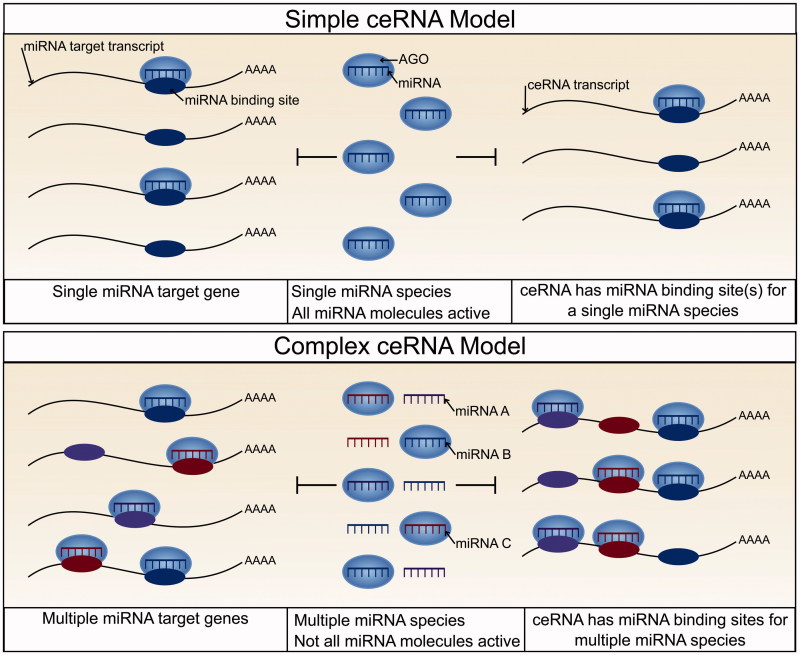

## Introduction

**Figure 1.**
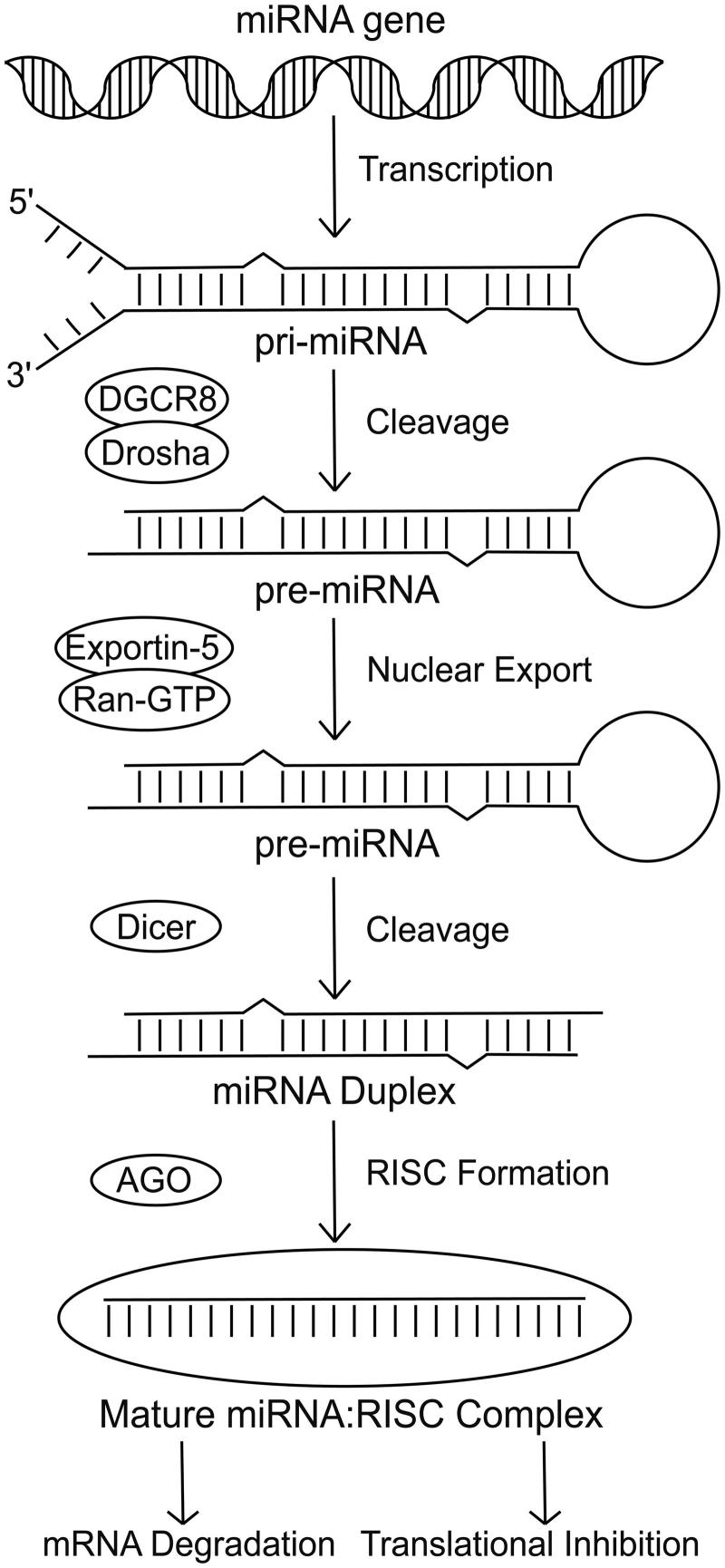
Pathway of miRNA biogenesis. The canonical pathway of miRNA biogenesis initiates with transcription of the miRNA sequence to form the pri-miRNA. The pri-miRNA is then cleaved by the microprocessor complex (Drosha-DGCR8) to form a hairpin precursor termed the pre-miRNA. Exportin-5-Ran-GTP exports the pre-miRNA from the nucleus into the cytoplasm where it is further cleaved by Dicer. The functional strand of the mature miRNA is then incorporated into an Argonaute protein as part of the RNA-induced silencing complex. This complex is then able to target mRNAs and repress them via a mechanism of mRNA degradation or translational inhibition.

RNA and protein abundance is regulated by transcription and translation, as well as by the turnover and processing of both messenger RNA (mRNAs) and proteins. Although most studies focus on the transcriptional control of gene expression, the importance of post-transcriptional regulation in cellular homeostasis is becoming increasingly clear. MicroRNAs (miRNAs) are key modulators of post-transcriptional regulation and have been implicated in stress responses and human disease (Leung and Sharp 2010; Mendell and Olson 2012). These are small, ∼22 nucleotide, non-coding RNAs (Bartel 2004) that when incorporated into a member of the Argonaute (AGO) family of proteins, as part of the RNA-induced silencing complex (RISC), bind to transcripts at sites sharing partial complementarity to that of the miRNA, and down-regulate expression (Figure 1) via a mechanism of mRNA degradation and/or translational inhibition (Valencia-Sanchez 2006). Approximately 60% of all human protein-coding transcripts are evolutionarily conserved targets of miRNAs (Friedman et al. 2008), suggesting that the role of miRNAs in post-transcriptional regulation is important, ancient, and widespread.

Recently, it was proposed that the repressive actions of miRNAs are themselves modulated by the pool of mRNAs that contain miRNA binding sites. Here, each additional miRNA binding site reduces the availability of the miRNA to other binding site containing transcripts, thereby reducing the extent to which these latter transcripts could be repressed. This mechanism is supported by evidence that artificially expressed mRNAs containing a high number of high affinity miRNA binding sites are indeed able to alter miRNA-mediated gene repression (Ebert et al. 2007). Furthermore in *Arabidopsis thaliana* a non-coding RNA, *ISP1*, was shown to sequester miR-399 thereby increasing the accumulation of other miR-399 target transcripts (Franco-Zorrilla et al. 2007). Such early evidence eventually led to the competitive endogenous RNA (ceRNA) hypothesis. This proposes that mRNAs that share binding sites for the same set of miRNAs can indirectly regulate one another’s cellular abundance through their competition for miRNA binding (Marques et al. 2011; Salmena et al. 2011). The ceRNA hypothesis, however, is controversial. The critical unresolved issue is whether physiological changes in the abundance of a miRNA’s target transcript are sufficient to substantially alter the abundance of other miRNA targets, particularly owing to the high abundance and diversity of target transcripts expected for each miRNA.

Here we focus on aspects of miRNA targeting and ceRNA crosstalk that we believe deserve further investigation, how they relate to our current understanding of the ceRNA mechanism, and how modeling of these molecular mechanisms could be improved. Our view is that, on balance, the experimental evidence is in favor of the notion that ceRNA crosstalk can be physiologically relevant. Further experimental evidence, however, clearly is required to enhance our understanding and to demonstrate the prevalence of such ceRNA crosstalk.

### Current models of ceRNA crosstalk

The stoichiometry between miRNAs and their target sites that is able to promote ceRNA crosstalk has been investigated using mathematical models. These were created assuming a titration reaction among: (i) a transcript defined as the ceRNA, (ii) the mediating miRNA, and (iii) one or more other mRNAs targeted by the miRNA. Some studies conclude that ceRNA crosstalk has greatest effect when the ceRNA and miRNA target transcripts are expressed at equimolar concentrations, and when miRNAs are neither lowly nor highly abundant (Ala et al. 2013; Figliuzzi et al. 2013). Others conclude that ceRNA crosstalk is maximal when the abundances of the ceRNA and mediating miRNA are equimolar (Hausser and Zavolan 2014) or when the ceRNA effectively doubles the number of miRNA target sites (Jens and Rajewsky 2014). These contrasting conclusions appear to depend on the model used, and include variables such as the numbers of ceRNAs, miRNAs, and miRNA targets that are considered, and whether the miRNA is released intact or degraded following target repression. Importantly, most of these mathematical models assume that the number of miRNA molecules exceeds the number of target sites, which is counter to what has been shown experimentally (Bosson et al. 2014). These mathematical models thus have not resolved the question of the relative abundances of various RNA species required to permit physiological ceRNA crosstalk.

**Figure 2.**
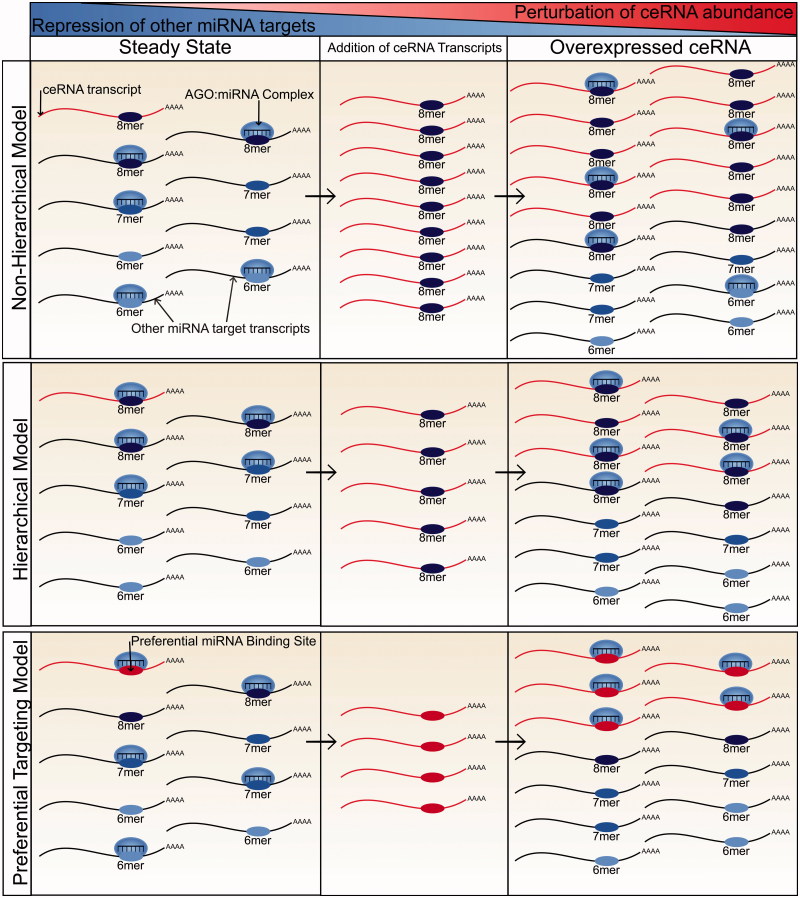
A comparison of models of miRNA targeting and how each relates to the potential for ceRNA crosstalk. In the nonhierarchical model, miRNA molecules bind target transcripts independently of their affinity for their miRNA binding sites. As a result, a ceRNA has to contribute an equivalent number of miRNA binding sites to those already present in the transcriptome before significant derepression of endogenous miRNA target transcripts will be observed. Due to such a high requirement for additional miRNA binding sites, the potential for ceRNA crosstalk is low. In the hierarchical model, miRNA molecules preferentially bind higher affinity sites (8mers) before spreading across low affinity sites. A ceRNA with a high affinity miRNA binding site therefore only has to contribute miRNA binding sites at a number similar to the miRNA molecule count before significant derepression of targets will be observed. Therefore, there is potential for ceRNA crosstalk provided that the miRNA is not highly abundant in comparison to the number of its high affinity binding sites. In the preferential targeting model, certain transcripts are preferentially targeted and repressed by miRNA molecules. In this model, the potential for ceRNA crosstalk is high if the ceRNA is a preferentially targeted transcript. However, it is currently unclear what factors may contribute to preferential targeting (see color version of this figure at www.tandfonline.com/ibmg).

The stoichiometric relationships among miRNAs and their targets have also been investigated experimentally although with contrasting results (Figure 2). Bosson et al. (2014) suggested that miRNAs bind their target mRNAs hierarchically, preferentially binding to rare high affinity target sites over the more abundant, lower affinity sites. This hierarchy effectively reduces the pool of available miRNA target sites with the consequence that a ceRNA with high affinity miRNA binding sites has to contribute fewer such sites in order to cause derepression of other miRNA-targeted transcripts. Through the use of reporter genes, the study then showed that a ceRNA can contribute sufficient miRNA binding sites to derepress other miRNA targets when its abundance lies within a physiological range, but only for miRNAs with a low or intermediate miRNA:target ratio.

A contrasting model was proposed by Denzler et al. (2014, 2016), which suggests that the spread of miRNA binding across target transcripts is independent of the affinity of the miRNA binding sites. They show that a ceRNA has to contribute a similar, and thus very large, number of miRNA binding sites to those already present in the transcriptome if it is to alter the repression of miRNA targets. The authors focused on miR-122, the most abundant liver miRNA (Tang et al. 2011; Ludwig et al. 2016), to show that no transcript, or collective changes in transcript abundance, could contribute a sufficiently high number of additional binding sites to alter miR-122 target repression (Denzler et al. 2014). They then extrapolated from these findings to conclude that ceRNA crosstalk is not possible within a physiological range of transcript abundance.

Mathematical models and experimental results thus provide no consensus as to whether ceRNA crosstalk can occur under physiological cellular conditions.

### Evidence supporting the ceRNA hypothesis

Despite the controversy over the physiological relevance of ceRNA crosstalk, there are a growing number of transcripts that have been proposed to act as ceRNAs. The first experimentally supported mammalian ceRNA was that of *PTENP1*, a transcribed pseudogene which regulates the mRNA and protein abundance of the tumor suppressor gene *PTEN* (Poliseno et al. 2010). It does this in a miRNA-dependent manner owing to its sharing of multiple conserved miRNA binding sites with *PTEN*. *PTENP1* was further shown to have a suppressive role in cell proliferation and is selectively lost in human cancer (Poliseno et al. 2010). Since then, many mRNAs (Jeyapalan et al. 2011; Sumazin et al. 2011; Tay et al. 2011; Gao et al. 2016), lncRNAs (Wang et al. 2010; Cesana et al. 2011; Johnsson et al. 2013; Wang et al. 2013; Tan et al. 2014, 2015), pseudogene transcripts (Marques et al. 2012; Karreth et al. 2015; Ye et al. 2015; Straniero et al. 2017), and circular RNAs (Hansen et al. 2013; Memczak et al. 2013) have been suggested to act as ceRNAs. Many of these diverse transcripts have proposed roles in human disease including in various types of cancer (Wang et al. 2010; Jeyapalan et al. 2011; Sumazin et al. 2011; Tay et al. 2011; Johnsson et al. 2013; Karreth et al. 2015; Ye et al. 2015; Gao et al. 2016) and in neurodegenerative diseases (Tan et al. 2014; Straniero et al. 2017). It is also proposed that ceRNAs modulate the differentiation of embryonic stem cells (Wang et al. 2013; Tan et al. 2015). Unfortunately, many of these studies fail to provide substantial evidence of physiological effects that are explicable by a ceRNA mechanism. For example, some studies do not demonstrate that the effects of a potential ceRNA are miRNA-dependent, or else fail to address whether the number of additional binding sites provided by the *ex vivo* overexpression of a ceRNA exceeds the number achievable under physiological levels of gene expression. An exception is a recent investigation of *CDR1as*, a circular RNA which is highly expressed in the mouse brain and contains >70 binding sites for miR-7. Removal of this locus in mice disrupted miR-7-mediated gene repression, altering mRNA abundance by up to 2-fold and leading to dysfunction of neuronal activity (Piwecka et al. 2017). This study provided the first *in vivo* evidence of a functional circular RNA and of a physiologically relevant ceRNA mechanism in mammals.

### Formation and activity of the miRNA:RISC complex

**Figure 3.**
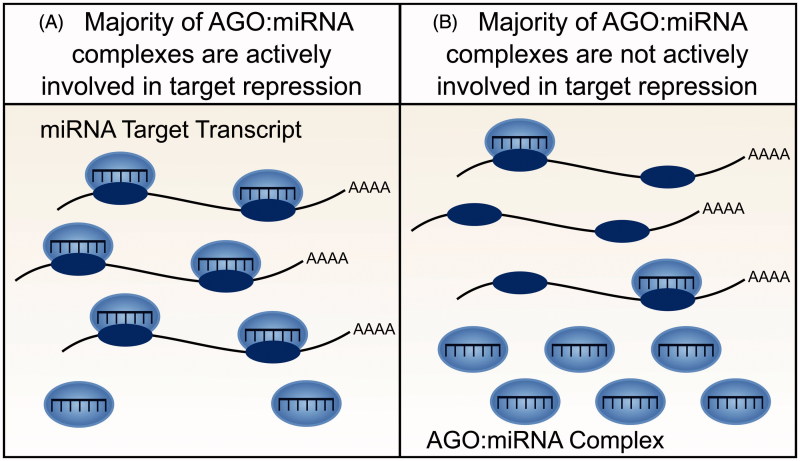
Availability and activity of miRNA molecules. Not all miRNA molecules present within a cell are active and available for target gene repression. (A) In cell lines, for example, the majority of AGO:miRNA complexes are actively involved in targeting and repression (La Rocca et al. 2015). (B) In contrast, within tissues, the majority of AGO:miRNA complexes are inactive (La Rocca et al. 2015). The effect of a ceRNA will depend on the number of active AGO:miRNA complexes, with greater crosstalk predicted when a smaller number of AGO:miRNA complexes are active (see color version of this figure at www.tandfonline.com/ibmg).

Resolution of the ceRNA controversy requires a better understanding of the molecular specificity and dynamics of miRNA-mediated target repression. Several mathematical models of ceRNA action (as introduced above) require miRNA molecules to outnumber target sites. Furthermore, both mathematical and experimental models additionally assume that cells contain an aqueous solution wherein all miRNA, RISC, and target transcript molecules diffuse freely, are active and are fully available for interaction. However experimental observations imply that these assumptions are violated. In particular, the repressive effect of a miRNA cannot be accurately predicted from its cellular abundance alone (Mullokandov et al. 2012). A miRNA’s association with RISC is a better indicator of miRNA activity (Flores et al. 2014) yet only a small proportion of miRNA:RISC complexes have been shown to be actively engaged in target repression in adult tissues (La Rocca et al. 2015). Conversely, in cell lines the majority of miRNA:RISC complexes are involved in target repression, which highlights an important distinction between cell lines and the adult tissues that they represent (Figure 3) (La Rocca et al. 2015). Finally, models of ceRNA crosstalk do not account for recent unexpected observations that the association of a miRNA to RISC can be modulated by the number of high affinity mRNA targets of this miRNA:RISC complex (Flores et al. 2014). These findings indicate, first, that not all miRNA molecules within a cell are involved in active repression of target transcripts and, second, that the repressive action of a miRNA can be altered through its activity, independent of changes in miRNA abundance.

Such discoveries have an important implication for ceRNA models: if the proportion of active miRNA molecules in a cell is small then there is an increased likelihood that changes in the abundance of a ceRNA transcript will alter its interaction with sufficient miRNA molecules to affect the extent of repression of other target transcripts.

### Heterogeneity of miRNA targets, target sites, and binding

Modelling ceRNA crosstalk becomes increasingly complex in light of the fact that ceRNAs will bind and sequester miRNAs with unequal efficiency. Any investigation of how the number of additional miRNA binding sites contributed by a ceRNA influences the repression of other miRNA targets will therefore be relevant to that ceRNA only. Here we discuss processes that alter the efficiency of miRNA targeting and repression, thereby altering the potential effectiveness of a ceRNA.

**Figure 4.**
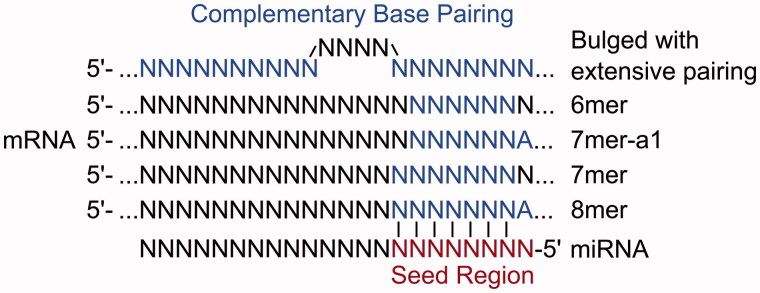
Alternate types of miRNA binding sites. Each site type has a different affinity based upon the extent of base pairing to the miRNA (see color version of this figure at www.tandfonline.com/ibmg).

**Figure 5.**
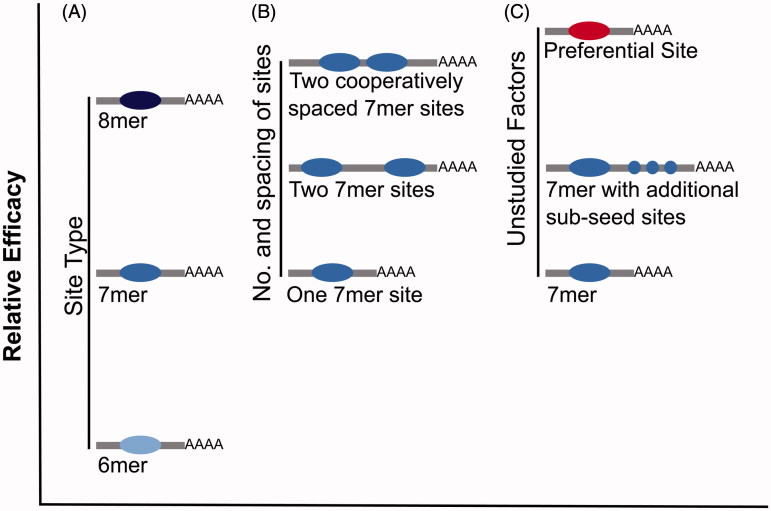
The relative efficacy of miRNA-mediated repression of various site types. It is hypothesized that sites with a greater efficacy of miRNA binding and repression also show a greater efficacy for ceRNA crosstalk. (A) Relative efficacy of canonical site types. (B) Relative efficacy of a single site, versus two sites or two cooperatively spaced sites. (C) Hypothesized efficacy of unstudied site types (e.g. preferential binding sites and additional sub-seed sites) versus a canonical 7mer site (see color version of this figure at www.tandfonline.com/ibmg).

An important factor in determining the recognition of miRNA targets, the efficacy of repression and the potential for a transcript to act as a ceRNA, is the extent of base pairing between the 3′-UTR of a mRNA and nucleotides 2–8 at the 5′ end of the miRNA, termed the miRNA seed region (Lewis et al. 2005). The most effective canonical site types are the 8mer site (base pairing to nucleotides 2–8 of the miRNA with an A opposite nucleotide 1), followed by 7mer sites (base pairing to nucleotides 2–8 of the miRNA or nucleotides 2–7 with an A opposite nucleotide 1) and the much weaker efficacy 6mer sites (base pairing to nucleotides 2–7 of the miRNA) (Lewis et al. 2005) (Figure 4). As these site types determine the effectiveness of miRNA repression, they are also expected to determine the effectiveness of a miRNA binding site containing transcript to act as a competitor (Figure 5(A)). The number of additional binding sites that a ceRNA needs to contribute before derepression is observed for other miRNA target transcripts is variable, differing by site type. One study reported that 7mer sites are 50% as effective, and 6mer sites are 20% as effective, as 8mer sites (Denzler et al. 2016). ceRNAs containing high affinity miRNA binding sites should thus be more efficient at crosstalk.

miRNA binding sites with extensive complementarity to the miRNA are the most effective, of the site types currently tested, in causing derepression of miRNA targets, being approximately 4-fold more effective than 8mer sites (Denzler et al. 2016). Binding to such highly complementary sites tends to trigger miRNA degradation (Ameres et al. 2010), implying that this causes derepression of miRNA target genes primarily through a reduction in miRNA activity rather than competition for binding. Interestingly, the effectiveness of any ceRNA containing such highly complementary miRNA binding sites depends on the initial abundance of the miRNA, and not on the abundance of competing miRNA binding sites, in contrast to the model of competition proposed for canonical 8–6mer miRNA binding sites (Denzler et al. 2016). Although transcripts containing such an extensively paired miRNA binding site have an increased potential as a ceRNA, such sites are thought to be rare in mammals (Bartel 2009) and therefore are unlikely to contribute substantially to ceRNA crosstalk.

In addition to miRNA binding site complementarity, the number and location of miRNA binding sites are also important factors for explaining the variable efficacy of miRNA-mediated repression (Doench et al. 2003; Grimson et al. 2007) and the potential for a miRNA target transcript to act as a ceRNA (Figure 5(B)) (Denzler et al. 2016). A miRNA target transcript that contains more miRNA binding sites will cause a larger change in total site count when its abundance is altered, and thus it will have a greater potential to act as a ceRNA when expressed within a physiological range of gene expression. The effectiveness of these multiple sites can also be altered by their spacing. Conserved miRNA binding sites tend to be separated by 10–130 nucleotides (Saetrom et al. 2007), with 8–40 nucleotides demonstrated experimentally to be optimal (Grimson et al. 2007; Saetrom et al. 2007). It is expected that miRNA binding sites separated by fewer than 8 nucleotides are less likely to be simultaneously occupied due to steric hindrance between adjacent miRNA:RISC complexes. Why sites within 40 nucleotides of each other act more cooperatively is, however, less clear perhaps being due to complex formation at one site either actively recruiting or aiding in the stabilization of another.

Proximal miRNA binding sites have been shown to act cooperatively in ceRNA crosstalk. A reporter gene was created containing a miRNA binding site for let-7 and a miRNA binding site for miR-122 separated by 58 nucleotides. Derepression of endogenous targets of these miRNAs was then shown to require the addition of 20–50% fewer reporter transcripts than when separated miRNA binding sites were tested (Denzler et al. 2016). Interestingly, this effect was observed for endogenous transcripts targeted by both of these miRNAs, as well as endogenous transcripts targeted by only one. Two observations are of particular note here. First, a transcript with multiple cooperatively spaced miRNA binding sites appears to have a greater potential as a ceRNA. Second, binding of multiple miRNA species to a ceRNA could occur synergistically, so that the presence of a cooperative binding site for one miRNA can influence competition for binding of an alternate miRNA. Thus, the potential of a transcript to act as a ceRNA may depend on the total number and identity of all miRNA binding sites not just the number of binding sites present for a particular miRNA.

These are the currently known factors that affect not just the efficacy of repression of a miRNA target but also the potential for that target to act as a ceRNA. Transcripts that are most effective at competing for miRNA binding are expected to contain a large number of high affinity miRNA binding sites with some degree of optimal spacing. The potential for ceRNA crosstalk has been tested for only a relatively small number of reporter transcripts, each typically containing 1–3 cooperatively spaced high affinity miRNA binding sites (Bosson et al. 2014; Denzler et al. 2014, 2016), and hence these may not represent the most effective ceRNAs. Similarly, as endogenous targets of miRNAs are unequal in their efficacy of repression, studies that employ an endogenous miRNA target may not be investigating an effective ceRNA. Consequently, despite the conclusions of others (Denzler et al. 2016), it remains plausible that ceRNA crosstalk occurs within a physiological range of gene expression but only for a subset of transcripts that are distinguished by their efficiency at recruiting and binding miRNAs. In support of such effective transcripts, Werfel et al. (2017) demonstrated that some miRNA target transcripts are preferentially bound and repressed by miRNAs. Furthermore, they found that these targets are neither enriched for a higher number of miRNA binding sites, nor have particularly high expression levels, suggesting that there are unstudied factors that enhance miRNA binding (Figure 5(C)). For future studies of ceRNA crosstalk, it may therefore be more instructive to utilize either endogenous transcripts that have already been proposed to act as a ceRNA, or a reporter transcript based on such a ceRNA. Alternatively, the approach used in Werfel et al. (2017), using RNA-seq following miRNA inhibition and Argonaute-2 (AGO2)-RNA immunoprecipitation (RIP), could be used to determine the miRNA target transcripts with the greatest level of miRNA binding, and thus the greatest potential as a ceRNA. Derepression of endogenous targets should also be studied on a target-by-target basis, because some targets are likely to be more susceptible than others to ceRNA-mediated derepression.

### Dynamics of miRNA targeting and repression

In order to repress miRNA target genes the miRNA:RISC complex has first to be efficient at encountering miRNA targets from among the complex pool of cellular RNAs, and then to bind these targets with sufficient affinity to mediate repression. The mechanisms by which miRNA:RISC complexes are able to efficiently engage with target sites are poorly understood, but are thought to involve both diffusion through the cytoplasm and lateral diffusion along RNA transcripts, similar to the facilitated diffusion mechanism initially proposed for transcription factors searching for DNA target sites (Berg et al. 1981). Diffusion of a miRNA:RISC complex through the cytoplasm is slower than lateral diffusion across an RNA transcript, yet would allow miRNA:RISC complexes to sample a greater proportion of binding sites. Therefore, a mixture of these two diffusion processes appears to be vital for efficient miRNA targeting. Single molecule fluorescence studies show that miRNA:RISC complexes use lateral diffusion to sample multiple binding sites along the length of a target RNA with greater than 90% of initial miRNA:RISC binding events being resolved by shuttling to an alternate target site (Chandradoss et al. 2015). Although the target search is more effective using both long distance and local diffusion, the speed of the search process is in conflict with the specificity of binding: the more stable the binding of the miRNA:RISC complex to a target, the more stable the binding will be to similar off-target sequences thus slowing the target search. To be efficient, a miRNA:RISC complex should therefore initiate its search for a target using a low affinity binding strategy before switching to a repressive mode in which binding to the target site is of higher affinity (Klein et al. 2017).

Recent evidence in support of this hypothesis suggests that human AGO2 recognizes target sites in a step-wise manner: nucleotides 2–5 (the sub-seed region) of the miRNA are first exposed for base pairing with the target before a conformational change permits further bonds to form between the miRNA seed region and the target site (Schirle et al. 2014). It is this sub-seed region of the miRNA that is used for initial screening of target sites (Chandradoss et al. 2015; Salomon et al. 2015). Only when additional nucleotides of the miRNA are exposed for base pairing with the target is the level of stability sufficient to permit repression of its target transcript.

Consequently, an increased density of sub-seed sites on a miRNA target site-containing transcript may increase the efficiency by which that transcript is targeted and may modulate the efficacy of a ceRNA independently of its number of full seed-matching target sites. If so, then this would alter how we currently assess the potential of a transcript to act as a ceRNA because, in one model, for a ceRNA to exhibit effective crosstalk it needs to contribute an equivalent number of seed-matched target sites to those already present within the transcriptome (Denzler et al. 2016).

Binding affinity is not the only factor determining miRNA-mediated target repression. The distribution of miRNA:RISC across target sites is also regulated by phosphorylation of AGO2 (Golden et al. 2017). Binding of AGO2 and an associated miRNA to a target site induces AGO2 phosphorylation which then promotes its dissociation from the target site. Loss of AGO2 phosphorylation impairs miRNA-mediated gene repression and dramatically expands the number of target sites bound to AGO2:miRNA under steady-state conditions, showing that under normal conditions AGO2:miRNA complexes target only a subset of the potential target pool. Interestingly, some target transcripts retain the ability to be bound by AGO2 when it is unable to be phosphorylated, despite the greatly expanded target pool. It is possible that these preferentially bound targets could represent transcripts that are highly efficient at recruiting and binding miRNAs and would make good candidate ceRNAs. These targets contain a mixture of higher affinity 8mer sites, as well as lower affinity 7mer and 6mer target sites. Thus, it is not affinity of the miRNA binding site that determines the ability of a transcript to retain miRNA binding upon expansion of the target pool. Indeed, the only difference observed between transcripts that retained miRNA binding upon loss of AGO2 phosphorylation and transcripts that lose both miRNA binding and repression was the rate of transcript decay, with slower decay rates associated with the preferentially bound transcripts. This result suggests that the AGO2 phosphorylation cycle is a timing mechanism that limits the residency time of AGO:miRNA:target interactions.

Nevertheless, how this AGO2 phosphorylation cycle promotes efficient miRNA-mediated repression remains unclear. It is possible that conformational changes of AGO2 upon target binding trigger AGO2 phosphorylation thereby limiting the residency time of the AGO2:target interaction. Alternatively, additional transcript features, such as sites for RNA binding proteins, may specifically promote AGO2 phosphorylation, and thus AGO2:target dissociation (Golden et al. 2017). Whatever the mechanism, it now appears that AGO2:miRNA complexes typically target only a subset of all possible targets in the transcriptome. An important consequence of such a reduction in effective miRNA binding site number is that, theoretically, it enhances the potential for ceRNA crosstalk, provided that the ceRNA is one of the subset of transcripts that are efficiently targeted by the miRNA.

### Subcellular localization

**Figure 6.**
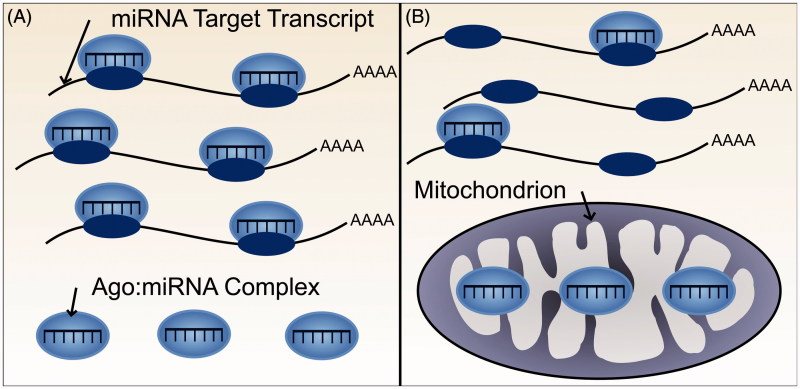
Subcellular localization of miRNAs, and other components of the miRNA silencing pathway could alter the extent of miRNA-mediated repression and thus potential for ceRNA crosstalk. (A) Both miRNA target transcripts and AGO:miRNA complexes are localized throughout the cytoplasm. The miRNA, therefore, is able to bind and repress its target transcripts. (B) The miRNA target transcripts are localized throughout the cytoplasm but AGO:miRNA complexes are predominantly localized elsewhere, for example, within mitochondria. Consequently, miRNA-mediated repression of the target transcript would be minimal (see color version of this figure at www.tandfonline.com/ibmg).

Efficient miRNA-mediated repression requires cellular co-localization of the interacting components of the silencing pathway: miRNAs, components of the RISC and miRNA target transcripts (Figure 6). As pre-miRNAs are processed into mature miRNAs within the cytoplasm it is likely that the majority of miRNA:target interactions also occur there, although whether these interactions typically occur diffusely throughout the cytoplasm or within specific cytoplasmic locales remains unclear. Here we discuss evidence that miRNAs localize to many of the subcellular compartments of the cytoplasm, such as processing bodies and several cellular organelles, as well as the potential functions of this subcellular compartmentalization.

AGO2 is localized to discrete cytoplasmic foci termed cytoplasmic bodies or processing bodies (P-bodies; Sen and Blau 2005) that contain factors involved in the RNA decay process (Cougot et al. 2004) including many components of the miRNA silencing pathway such as miRNAs (Liu et al. 2005), their target transcripts (Liu et al. 2005) and the AGO-interacting protein GW182 (Eystathioy 2002). Although AGO2 is found at a much higher concentration in P-bodies than the surrounding cytoplasm, only around 1% of AGO2 is actually localized within P-bodies (Leung et al. 2006) and P-body formation is not required for normal miRNA-mediated repression and degradation of target mRNAs (Eulalio et al. 2007), suggesting that most miRNA activity occurs elsewhere in the cell.

Other potential locations for miRNA-mediated gene silencing are cellular organelles, such as the endoplasmic reticulum (ER). Although major components of RNA-mediated gene silencing are found in many subcellular compartments and throughout the cytoplasm, miRNAs which are incorporated into AGO2 predominantly co-sediment with membranes of the rough ER (Stalder et al. 2013). In one model (Stalder et al. 2013), loading of the miRNA into AGO2 and interaction of the RISC with a target mRNA co-occur at the ER’s cytosolic membrane surface, allowing highly efficient repression particularly of translating mRNAs containing miRNA binding sites.

Some miRNAs (Bandiera et al. 2011; Barrey et al. 2011; Zhang et al. 2014), as well as AGO2 (Bandiera et al. 2011; Zhang et al. 2014), are localized to mitochondria. In the undifferentiated myoblast cell line C2C12 approximately 13% of total AGO2 localized to mitochondria (Zhang et al. 2014). However, miRNA-mediated repression in mitochondria is in doubt because it apparently lacks GW182, a RISC component required for miRNA-mediated repression (Zhang et al. 2014). Furthermore, although interactions have been reported between AGO2 and mitochondrial genome-encoded mRNA targets, some miRNAs are reported to have opposing effects, specifically on COX1 abundance (Das et al. 2012; Zhang et al. 2014) with one miRNA enhancing translation of COX1 in sharp contrast to the canonical role for miRNAs (Zhang et al. 2014).

Most studies examining the cellular localization of miRNAs and other components of the RNA-mediated silencing pathway have been performed under steady-state conditions. The fluctuating concentrations of these components throughout the cell over time, or following cellular stimuli, are thus largely unknown. Nevertheless, it is likely that local changes in miRNA, miRNA target transcript and RISC component levels influence miRNA activity. In neurons, stimulation of a single synapse caused increased processing of a pre-miRNA into mature miRNA, which in turn resulted in decreased protein synthesis of a target mRNA in a spatially restricted manner (Sambandan et al. 2017). The spatiotemporal dynamics of miRNA:target interactions therefore are likely complex. ceRNAs may exhibit enhanced crosstalk within subcellular compartments in which it need not compete for miRNA binding with all other target transcripts present elsewhere in the cell.

### miRNA-to-target ratio and the potential for ceRNA crosstalk

The effect of miRNA-to-target ratio on the repression of miRNA target transcripts is relatively well understood: a miRNA with a large number of targets, relative to its own abundance, typically shows a weaker repression of its targets than a miRNA with a smaller target abundance (Arvey et al. 2010). Thus, only highly expressed miRNAs, those that are more abundant than their high affinity (e.g. 8mer) target sites, show appreciable binding to these sites and confer repressive activity (Bosson et al. 2014). Even these active miRNAs are typically not expressed sufficiently to bind to the large number of lower affinity sites (e.g. 6mers) above background rates (Bosson et al. 2014), which may begin to explain the lower efficacy of repression of these sites (Grimson et al. 2007).

The miRNA-to-target ratio’s effect on ceRNA crosstalk potential is less clear and has led to two opposing models of ceRNA crosstalk (as discussed above). A key difference in the models proposed by Bosson et al. (2014) and Denzler et al. (2014) is the effect that miRNA abundance has on the potential for ceRNA crosstalk. In the hierarchical model proposed by Bosson et al. (2014), a miRNA’s abundance determines its spread across the total pool of target sites. Consequently, it is miRNA abundance that determines the size of the effective target pool and thus the number of sites with which a ceRNA’s sites compete. By contrast, in the nonhierarchical model proposed by Denzler et al. (2014) the potential for ceRNA crosstalk is relatively unaffected by miRNA abundance, provided that the number of target sites is in excess of the number of miRNA molecules. When tested experimentally, the number of additional miRNA binding sites required to cause derepression of a miRNA target reporter showed little change upon increased or decreased miRNA abundance (Denzler et al. 2016), in line with the nonhierarchical model. However, this has only been demonstrated for a small number of miRNAs. Furthermore, it has been assumed that altering the abundance of miRNA molecules similarly alters the abundance of miRNA:RISC complexes that are actively involved in targeting, which may not be the case (Mayya and Duchaine 2015).

Overall, it is difficult to reconcile these two models (Bosson et al. 2014; Denzler et al. 2014) and draw a comprehensive conclusion regarding the effect of miRNA:target ratio upon the potential for ceRNA crosstalk. Both studies investigated the potential for ceRNA crosstalk using similar reporter constructs for the same miRNAs and in the same cell lines. Nevertheless, substantial differences were observed in the number of additional miRNA binding sites required to observe target gene derepression (Table 1). Interestingly, the conclusions drawn regarding the physiological relevance of ceRNA crosstalk by both Bosson et al. (2014) and Denzler et al. (2014, 2016) rely on assumptions that are at odds with experimental observations. Specifically, they have not accounted for the unequal efficiency of miRNA target transcripts containing the same number and affinity of binding sites to bind miRNA. As we discuss above, targets with a similar number and affinity of binding sites for one miRNA may exhibit contrasting levels of miRNA binding due to presence of sub-seed sites or clustered binding sites for an alternate miRNA. Consequently, some miRNA target transcripts will be more effective than others at competing for miRNA binding, thereby increasing the potential for these transcripts to act as ceRNAs.

**Table 1.** Summary of key differences between two models in regard to the effect of miRNA:target ratio upon ceRNA crosstalk.

	Bosson et al. (2014)	Denzler et al. (2016)
Model proposed	Hierarchical model where AGO:miRNA complexes are predominantly bound by high affinity target sites	Non-hierarchical model where AGO:miRNA complexes are evenly distributed across all target sites, independent of their affinity
Potential for ceRNA crosstalk	Defined by the ratio of the abundance of miRNA molecules to the number of their high affinity binding sites	Defined by the abundance of miRNA binding sites in the transcriptome
Method of defining the number of additional miRNA binding sites required for target derepression	Data grouped into bins by number of miRNA binding sites added. Derepression threshold defined as the lowest bin at which significant target derepression was observed	Derepression threshold defined as the point at which targets were derepressed by 10% of the total repression observed when no additional binding sites were present
Number of additional miRNA binding sites required for target derepression	miR-294: No derepression observed at 10,800 additional sites	miR-294: 22,000 additional sites
in mouse embryonic stem cells	miR-293: 3000 additional sites	miR-293: 9000 additional sites
	miR-92/25: 3000 additional sites	miR-92/25: 13,000 additional sites
Conclusions	ceRNA crosstalk is possible within physiological conditions provided that the miRNA:target pool ratio is low	ceRNA crosstalk is not possible within physiological conditions

The majority of studies investigating ceRNA crosstalk use a single ceRNA reporter transcript containing one or more miRNA binding sites for a single miRNA. Upon expanding our consideration to multiple ceRNAs, miRNAs, and miRNA target transcripts, the effects of abundance upon ceRNA crosstalk potential are even less well understood. The downstream consequences of increasing the abundance of a ceRNA that contains miRNA binding sites for multiple miRNAs are expected to be far more complex than those for a ceRNA with binding sites for just one miRNA. It is also possible that under certain conditions, for example during cellular differentiation or upon disease progression, multiple ceRNAs could be co-regulated thereby altering the abundance of miRNA binding sites more than is possible by an individual ceRNA. This issue of collective changes in transcript abundance was investigated for miR-122 in the liver, and transcriptome wide changes in a disease state were found to contribute an insufficient number of miRNA binding sites to cause observable derepression of other miRNA target transcripts (Denzler et al. 2014). Nevertheless, such transcriptome-wide effects on ceRNA crosstalk have not been investigated more broadly. It is therefore likely that the reporter systems in current use represent a simplistic form of ceRNA crosstalk that do not reflect the more complex physiological state.

### Strategies to identify and characterize a ceRNA mechanism

An increasing number of publications propose transcripts as ceRNAs. Nevertheless, most provide insufficient evidence to demonstrate conclusively a physiologically relevant ceRNA mechanism. Here we consider what evidence is required to identify and characterize a ceRNA (Table 2), and discuss how characterized ceRNAs could assist in improving the modeling of ceRNA crosstalk.

**Table 2.** Possible methods for identifying and characterizing a ceRNA.

Steps to identify a ceRNA	Possible methodology	Advantages and limitations of methodology
Identify a positive correlation in expression for a candidate ceRNA and transcripts with which it shares one or more miRNA binding sites	Use of existing expression datasets, e.g. GTEx, EMBL-EBI	Differences in gene expression may occur between the tissue type of interest and cell lines used for further experimental characterization of a ceRNA
	Analysis of gene expression in tissues/cells of interest, e.g. qRT-PCR, RNA-seq	Well-established experimental techniques
	Analysis of miRNA binding sites predicted computationally, e.g. TargetScan, miRanda	miRNA binding site prediction algorithms suffer from high rates of both false positive and false negative predictions
Alter abundance of candidate ceRNA and observe the effect upon abundance of other miRNA target transcripts	Increase abundance via an overexpression plasmid	May produce non-physiologically high levels of gene expression System is flexible and can be used to overexpress particular transcript (including mutated) isoforms, or the 3′-UTR alone
	Decrease abundance via shRNAs/siRNAs	Known off-target effects May alter availability of AGO2
	Increase/decrease abundance via CRISPRa/CRISPRi	Cannot differentiate between transcripts sharing promoter regions. CRISPRi may cause unintended transcriptional repression due to heterochromatin spread
Confirm miRNA-dependence of ceRNA crosstalk	Alter ceRNA abundance in Dicer knockout cells	Dicer knockout lines not available for many cell types
	Mutagenize miRNA binding site(s) on the ceRNA, e.g. site directed mutagenesis, CRISPR	More applicable to certain cell types depending on chromosome copy number and ability of cells to survive selection process
Confirm direct binding of miRNA to ceRNA and other target transcripts	Pulldown using biotinylated miRNA as bait	miRNA abundance cannot be kept at endogenous levels
	High-throughput RNA:RNA interaction assays, e.g. CLASH, CLIP	Low sensitivity: not all miRNA:target interactions will be identified
Confirm effects of ceRNA *in vivo*	Create mouse models with knockout of the proposed ceRNA and with a mutagenized miRNA binding site	Requires mouse orthologue miRNA binding sites, and the miRNAs involved, may not be conserved between human and mouse

An initial indicator that a transcript may act as a ceRNA under physiological conditions could be that its abundance is positively correlated with the abundance of transcripts that share binding sites for one or more miRNA species. Large datasets of physiological gene expression information such as GTEx (Lonsdale et al. 2013) will be useful for assessing positive correlation in gene expression across multiple samples from specific tissues. A second indicator could be that transcripts with positively correlated co-expression share an unexpectedly high density of miRNA binding sites predicted using computational algorithms such as TargetScan (Agarwal et al. 2015) and miRanda (Enright et al. 2003). These programs predict the presence of miRNA binding sites via the degree of transcript sequence complementarity to the miRNA seed region, along with other contextual factors such as predicted site accessibility and local AU content (Agarwal et al. 2015). However, *in silico* predictions of miRNA-target interactions suffer from high rates of both false positive (46–63%) and false negative (44–82%) predictions (Steinkraus et al. 2016). If seeking ceRNAs for a specific miRNA an alternative method could be to identify the strongest binding partner of that miRNA experimentally using the AGO-RIP method described in Werfel et al. (2017). Transcripts identified in this manner as preferential binding partners of a miRNA are hypothesized to be the most likely to be able to compete for miRNA binding when expressed within a physiological range.

Predicted ceRNA crosstalk requires experimental confirmation in the cell or tissue type of interest. Ideally experiments would be performed using tissue samples or primary cell lines; however, many experimental techniques are not possible in these model systems. Therefore, a cell line derived from the tissue of interest, which are easier to experimentally manipulate, may be the better choice as a model. As cell lines do not completely recapitulate the gene expression observed in the tissues they represent (Forrest et al. 2014), an important initial step will be to confirm the expression of any potential ceRNAs, their mediating miRNAs and the miRNA’s target transcripts. The ceRNA interaction can then be tested by first overexpressing the ceRNA, ideally within its physiological range of expression, and observing whether this leads to an increase in the abundance of transcripts targeted by the same miRNA(s), and then by observing the reciprocal relationship upon knockdown of the proposed ceRNA. However, the method chosen to alter abundance of the ceRNA may depend on the location of the ceRNA locus in the genome and whether the miRNA binding transcript also codes for protein. If the ceRNA is observed to alter a miRNA’s target transcripts’ abundance this should also be examined at the level of protein abundance for protein-coding genes. For a ceRNA to be functionally important the changes in abundance of other transcripts and proteins should be sufficient to perturb the activity of a cellular process. For example, in *Cdr1as* knockout mice, spontaneous vesicle release was up-regulated in neuronal cells (Piwecka et al. 2017).

Although the experimental workflow described above could identify potential ceRNAs and implicate their importance in a particular cellular process, it does not alone provide sufficient evidence that the altered cellular phenotype is mediated by a ceRNA mechanism. For this, the cellular effect needs to be shown to be miRNA-dependent, for example using Dicer knockout cell lines (where available) which are deficient in miRNA biogenesis: altering the abundance of a potential ceRNA in these Dicer-null cells should have no effect on other transcripts that share binding sites for the same miRNA(s). The identity of the specific miRNA that mediates ceRNA crosstalk should then be confirmed. If the potential ceRNA has been predicted to contain binding sites for multiple miRNAs, these can be tested systematically by altering the abundance of each miRNA in turn and then observing whether this affects the abundance of the ceRNA and other miRNA target transcripts.

Once mediating miRNAs have been identified, the predicted binding sites for these miRNAs should be mutated. It is expected that altering the abundance of the ceRNA will produce no effect upon gene expression or cellular processes when its binding sites for the mediating miRNA are abolished. Ideally, this miRNA binding site mutagenesis should also be performed on the endogenous transcript, perhaps via the use of CRISPR genome editing, to show that the presence or absence of the miRNA binding site has an effect on the abundance of other miRNA target transcripts when the ceRNA is expressed at endogenous levels. Direct binding of the mediating miRNA to the ceRNA and other target transcripts should also be demonstrated, for example via pulldowns using biotinylated miRNA as bait (Ørom and Lund 2007).

The final step to confirming that a transcript can act as a ceRNA is to demonstrate that a ceRNA mechanism that has been characterized *in vitro*, using the steps described above, is replicated *in vivo*. For example, if a human ceRNA has an orthologous sequence in mice, a mouse model could be created in which levels of the potential ceRNA are knocked down. Other targets of the mediating miRNA would be expected to be altered in abundance and, in turn, affect downstream physiological processes. The effect of abolishing the miRNA binding site on the ceRNA transcript should also be investigated *in vivo*. Lastly, in order to demonstrate that the abundance of the ceRNA transcript is responsible for altering cellular processes, rescue experiments could be performed with this model via the addition of the wild-type ceRNA transcript containing a functional miRNA binding site. If the ceRNA affects cellular homeostasis at a particular time point during development, however, rescue experiments performed at a later time point are likely to be ineffective.

The above lines of evidence should suffice to demonstrate convincingly that a transcript can alter the *in vivo* abundance and activity of other transcripts through a mechanism of ceRNA crosstalk. Any transcript conclusively shown to act as a ceRNA would be useful as a model for gaining a greater understanding of the crosstalk mechanism. It would be of interest to compare the number of copies of a transcript, and thus the number of additional miRNA binding sites, that are required to cause derepression of other miRNA target transcripts, specifically for a transcript identified to act as a ceRNA compared with other miRNA target transcripts with lower predicted potential as a ceRNA.

## Concluding remarks

Although the ceRNA hypothesis has provoked substantial interest, currently there is little conceptual concordance between studies modeling ceRNA mechanisms and others that propose specific ceRNA transcripts. This derives from our limited understanding of the factors affecting both miRNA-mediated repression and ceRNA crosstalk.

In this review, we propose that recently discovered aspects of miRNA targeting and efficacy of miRNA-mediated repression will also likely affect the potential for ceRNA crosstalk. We suggest that the field will need to take a more nuanced view of miRNA-mediated repression and ceRNA crosstalk, specifically by considering mechanistic models that are not solely based on the number and affinity of seed-matched target sites, but also account for altered RISC activity and subcellular molecular co-localization. Furthermore, understanding how stoichiometry between active miRNA:RISC complexes and miRNA target sites varies for different miRNA species or under different cellular conditions will be critical for demonstrating the relevance of ceRNA crosstalk as a physiological mechanism.

Similarly, in-depth characterization of *bona fide* ceRNAs may reveal factors that enhance crosstalk, such as presence of sub-seed and protein binding sites or specific sites of subcellular co-localization. While these factors remain obscure, and because they are likely to vary across different miRNAs and under variable cellular conditions, we suggest that any proposal that a transcript acts as a ceRNA should be considered according to its individual merits and available experimental evidence rather than whether it accords with a generalized theoretical model.
